# *In Vitro* Metacyclogenesis of *Leishmania (Viannia) braziliensis* and *Leishmania (Leishmania) amazonensis* Clinical Field Isolates, as Evaluated by Morphology, Complement Resistance, and Infectivity to Human Macrophages

**DOI:** 10.1155/2015/393049

**Published:** 2015-01-28

**Authors:** Ildefonso Alves da Silva, Camila Imai Morato, Valéria Bernadete Leite Quixabeira, Ledice Inácia de Araújo Pereira, Miriam Leandro Dorta, Milton Adriano Pelli de Oliveira, Maria Fátima Horta, Fátima Ribeiro-Dias

**Affiliations:** ^1^Instituto de Patologia Tropical e Saúde Pública, Universidade Federal de Goiás, Goiânia, GO, Brazil; ^2^Instituto Goiano de Oncologia e Hematologia, Goiânia, GO, Brazil; ^3^Departamento de Bioquímica e Imunologia, Instituto de Ciências Biológicas, Universidade Federal de Minas Gerais, Belo Horizonte, MG, Brazil; ^4^Setor de Imunologia, Instituto de Patologia Tropical e Saúde Pública, Universidade Federal de Goiás, Rua 235 S/N, Setor Universitário, 74605-050 Goiânia, GO, Brazil

## Abstract

This study was designed to assess *in vitro* metacyclogenesis of *Leishmania (Viannia) braziliensis* and *Leishmania (Leishmania) amazonensis* clinical field isolates obtained from patient lesions (*L. braziliensis* IMG3 and PPS6m; *L. amazonensis* MAB6). Metacyclogenesis was evaluated by different criteria, namely, promastigote size (morphometric analysis and flow cytometry), surface modifications (loss of lectin or monoclonal antibody (mAb) binding, complement resistance), and infectivity to human macrophages. Growth curves were similar for all parasites evaluated. The various features analyzed were expressed in a high percentage of promastigotes at 6th and 10th days of culture and a low percentage at the 2nd day. However, in most isolates, these features, considered as markers of metacyclogenesis, seemed to develop with different time courses, since the percentages of metacyclic forms detected with each technique were usually different. Parasites from 6th or 10th day and those negatively selected with lectin or mAb similarly infected human macrophages. From all isolates analyzed, *L. amazonensis* PH8 and MAB6 showed the highest and the lowest levels of susceptibility, respectively, to leishmanicidal activity of IFN-*γ*/LPS-activated macrophages. Our results showed that by using different techniques to evaluate different aspects of metacyclogenesis (morphological and biochemical modifications) different percentages of metacyclic promastigotes can be detected in each isolate culture.

## 1. Introduction

American tegumentary leishmaniasis (ATL) is mainly caused by* Leishmania (Viannia) braziliensis* and* L. (Leishmania) amazonensis* which lead to localized cutaneous leishmaniasis (LCL) but also can cause severe clinical forms as mucosal (ML) and diffuse cutaneous leishmaniasis (DCL), respectively [1–3]. The outcome of the disease depends on several aspects of the parasite-host relationship. Thus, isolating parasites from patient lesions and to studying the biological behavior of these clinical field isolates can allow the understanding of the parasite-host interactions.

Infection of hosts occurs by the injection of infective forms, known as metacyclic promastigotes, by parasite-harboring sand flies during blood meal. Metacyclic promastigotes develop inside the insect vector from procyclic promastigotes, less infective, which are newly transformed from amastigotes sucked with infected host cells. Metacyclogenesis can also occur in axenic cultures of parasites from the logarithmic to the stationary phase of growth [4–6]. Along with the increase of infectivity, metacyclogenesis includes changes in gene expression, morphology and biochemical structure of lipophosphoglycan (LPG) present in parasite surface [6–9]. In general, procyclic forms exhibit large cell body and short flagellum whereas metacyclic forms exhibit small body and a long flagellum usually with, at least, twice the cell body length [10]. Due to these alterations, it is possible to identify procyclic and metacyclic forms by morphometric analysis and flow cytometry [10, 11]. As opposed to procyclic forms, metacyclic promastigotes are resistant to complement-mediated lysis due to modifications in surface LPG and overexpression of the gp63 protease and probably to other proteins [7, 12–14]. Thus, using a complement resistance assay it is also possible to identify metacyclic forms in promastigotes cultures [15]. LPG carbohydrates expressed in procyclic forms can be detected by lectins or monoclonal antibodies (mAb) specific to these carbohydrates, which allow the identification, quantification, and isolation of metacyclic promastigotes by negative selection after lectin or the specific anticarbohydrate mAb treatment [5, 16–18].

Although metacyclogenesis has been well studied in* L. major* [5–7, 19] and New World* Leishmania* [10, 20, 21], this process has been poorly investigated in clinical field isolates of* L. braziliensis* and* L. amazonensis* [21]. The simultaneous use of techniques exploring different characteristics of metacyclic forms can allow us to better evaluate the process of metacyclogenesis of clinical field isolates of these species as well as to determine suitable experimental conditions to isolate the metacyclic promastigotes. Although humans and dogs are the most usual hosts for* Leishmania*, most studies evaluating infectivity of New World* Leishmania* promastigotes forms have used murine macrophages [11, 17, 18, 21, 22], with few using human macrophages [23–25]. Moreover, none of them has evaluated the metacyclogenesis of clinical field isolates or evaluated the infectivity of their metacyclic forms in human macrophage.

In the present study, we evaluated the* in vitro* metacyclogenesis of* L. (V.) braziliensis* and* L. (L.) amazonensis* clinical field isolates by parasite morphology, surface changes, and infectivity to human macrophages, comparing the results with those obtained from the well-studied* Leishmania* reference strains (*L. braziliensis* M2903;* L. amazonensis* PH8).

## 2. Material and Methods

### 2.1. Parasites and Cultures

Known strains of* L. braziliensis* (MHOM/BR/1975/M2903), named herein as M2903, and* L. amazonensis* (IFLA/BR/67/PH8), named herein as PH8, were used. The clinical field isolates were obtained from ATL patients with different clinical forms (LCL, ML, and DCL) and characterized as* L. braziliensis* and* L. amazonensis*, as we previously described [3, 26–28]. Two clinical field isolates of* L. braziliensis*, MHOM/BR/2003/IMG (IMG3), obtained from LCL lesion, and MHOM/BR/2006/PPS (PPS6m), obtained from ML lesion, and one isolate of* L. amazonensis* MHOM/BR/2006/MAB (MAB6), obtained from DCL lesion, were assessed.

Promastigotes were all stored in logarithmic phase of growth (2-3 days of culture) in liquid nitrogen (Leishbank/IPTSP/UFG, Goiânia, Goiás, Brazil), after differentiation from amastigotes derived from lesions of interferon gamma-knockout mice [28]. Cultures were made in 24-well plates (Costar, Cambridge, MA, USA) in Grace's Insect Medium (Sigma Chem. Co., St. Louis, MD, USA) supplemented with 20% fetal calf serum (FCS, Cripion, Andradina, SP, Brasil), 2 mM L-glutamine (Sigma), 100 U/mL penicillin (Sigma), and 100 *μ*g/mL streptomycin (Sigma), at 26°C, pH 5.8. Parasites were maintained in logarithmic phase of growth by passages each 2-3 days, always starting the cultures with 5 × 10^5^ parasites/mL. Only 3rd–6th passage parasites were used in the experiments.

Promastigotes growth curves were obtained by quantification of parasites after dilution of a culture aliquot in phosphate-buffered saline (PBS) containing 0.3% paraformaldehyde, in hemocytometer, during 12 days.

### 2.2. Metacyclogenesis Evaluation

#### 2.2.1. Morphometric Analyses

After 2, 6, or 10 day of culture, parasites were harvested and washed with PBS (1,000 g, 4°C, 15 min). The pellet was suspended in 5% FCS-containing PBS before centrifugation onto slides (3,500 g, 30 sec, cytocentrifuge Fanem, São Paulo, SP, Brazil). After staining (Instant Prov kit, Newprov, Pinhais, PR, Brazil), photomicrographs were taken under a light microscope (1,000x—5 images/slide). The images were analyzed using the software Image J (Atlanta, GA, USA). The cell body size and flagellum lengths were measured for 300 parasites in each day, and the percentage of metacyclic forms was calculated (promastigotes whose flagellum/body length ratio was ≥2 were considered metacyclic, according to [10]).

#### 2.2.2. Negative Selection with Lectin or Monoclonal Antibody

A previously described technique to identify and select the metacyclic promastigotes [11] was used. Briefly, parasites were collected from 2, 6, or 10 days of culture, washed with PBS (1,000 g, 4°C, 15 min), and suspended to 10^8^ parasites/mL in PBS.* L. braziliensis* and* L. amazonensis* suspension (300 *μ*L) were incubated with 100 *μ*g/mL* Bauhinia purpurea *lectin (BPL, Vector Laboratory, Burlingame, CA, USA), which binds to galactosyl (*β*-1,3) N-acetylgalactosamine (*β*-gal (1–3)-GalNAc) present in LPG of procyclic promastigotes or 100 *μ*g/mL 3A1-La mAb, which binds to LPG of procyclic promastigotes, but not of metacyclic forms [17, 18, 29], respectively. After 30 min, at room temperature (r.t.), cell suspensions were centrifuged (40 g, 5 min) and nonagglutinated metacyclic promastigotes were collected from supernatants. The metacyclic forms were immediately quantified and percentage of metacyclic forms was determined in relation to nonfractionated parasites.

#### 2.2.3. Flow Cytometry

Total parasites from 2nd, 6th, or 10th day of culture or negatively selected metacyclic forms (with BPL or 3A1-La mAb) were washed with PBS (1,000 g, 4°C, 15 min, 3x), quantified, and fixed with 1% paraformaldehyde in PBS (300 *μ*L) for flow cytometry analyses. Two thousand events were acquired using a FACScan Flow Cytometer (Becton Dickinson Immunocytometry Systems, San Jose, CA, USA) and parasites were analyzed after plotting the FSC × SSC [11] using the software Cyflogic (Turcu, PA, Finland) to identify procyclic (FSC^high^) and metacyclic (FSC^low^) forms.

#### 2.2.4. Complement Resistance Assay

Lyophilized rabbit serum (Cedarlane, Burlington, ON, Canada) was reconstituted in Hanks Balanced Salt Solution (HBSS, Sigma) and added to parasites at the concentration of 10% (from a dose-response curve previously assayed using M2903 parasites from 2, 6, or 10 days of culture). Parasites from 2nd, 6th, or 10th day of culture were collected and washed with HBSS (1,000 g, 4°C, 15 min). Parasites suspended in HBSS (5 × 10^5^) were incubated with 10% rabbit serum at 37°C, for 1 h. The suspension was kept at 4°C while quantification of live and dead parasites was carried out under a light microscope. Results were expressed as percentage of survived parasites, the complement resistant forms, in comparison with nontreated parasites.

### 2.3. Evaluation of Human Macrophage Infection

#### 2.3.1. Human Monocyte-Derived Macrophages

All procedures for macrophage derivation were approved by local Ethical Committee (Hospital das Clínicas/UFG, prot. 132/2012). Venous blood samples (~12 mL) were obtained from healthy blood donors (Blood Bank of Instituto Goiano de Hematologia e Oncologia, Goiânia, Goiás, Brazil) in EDTA-vacuum tubes (Greiner bio-one, Vacuette, Americana, SP, Brazil). Blood was layered on Ficoll gradient (GE Healthcare Bio-Sciences AB, Uppsala, Uppsala län, Sweden) and centrifuged at 1,000 g, r.t., 20 min. Mononuclear cells (5 × 10^5^ in 500 *μ*L RPMI 1640 medium supplemented with 10% FCS, 11 mM sodium bicarbonate, 2 mM L-glutamine, 100 U/mL penicillin and 100 *μ*g/mL streptomycin (all reagents from Sigma)), were distributed onto 13 mm-round glass coverslips (Knitel, Varrentrappstr, Bielefeld, Germany) and placed in 24-well plates (TPP, Techno Plastic Products, Trasadingen, Swizerland). Culture medium was replaced each 48 h during 7 days (36°C, 5% CO_2_). Nonadherent cells were removed during medium changes and adherent monocytes differentiated into macrophages. For some experiments, at the 6th day of culture cells were treated with 10 ng/mL of recombinant human IFN*γ* (Sigma) overnight, before macrophage infection.

#### 2.3.2. Macrophage Infection

Growth stationary phase promastigotes or metacyclic forms isolated by negative selection with BPL/3A1-La mAb were washed with PBS (1,000 g, 10°C, 15 min). Parasites were added to macrophage cultures (5 × 10^5^ parasites/well) at a multiplicity of infection (MOI) ~10 : 1 (considering that monocytes comprise ~10% of the mononuclear cells). Cultures preincubated with recombinant human IFN*γ* (10 ng/mL; 24 h) were treated with lipopolysaccharide (LPS; 50 ng/mL* E. coli* 0111:B4, Sigma) at the same time of parasite addition. Cultures were incubated for 24 h or 72 h, 36°C, 5% CO_2_. After incubation, cells were fixed and stained with commercial Instant Prov kit (Newprov) and analyzed under a light microscope (1,000x) to determine the infection index. Three to five hundred cells were analyzed in random fields and the percentage of infected cells and the mean number of intracellular amastigotes per infected cell (at least 50 infected cells were counted) were determined. Infection index = percentage of infected cells × mean number of parasites per infected cell. All assays were done in duplicate and cell infection was blinded evaluated.

### 2.4. Statistical Analysis

For each evaluation, 3 to 6 experiments in triplicates were performed. Data are shown as median with interquartile range (minimum and maximum) or mean ± EPM. Mann-Whitney, Wilcoxon matched pair or *t*-student test were used when suitable, and the level of significance was set at *P* < 0.05. The analyses were performed using GraphPad Prism 5.0 Software (San Diego, CA, USA).

## 3. Results

### 3.1. *In Vitro* Metacyclogenesis of Clinical Field Isolates of* L. braziliensis* and* L. amazonensis* as Evaluated by Morphological Alterations (Morphometry and Flow Cytometry)

**Figure 1:**
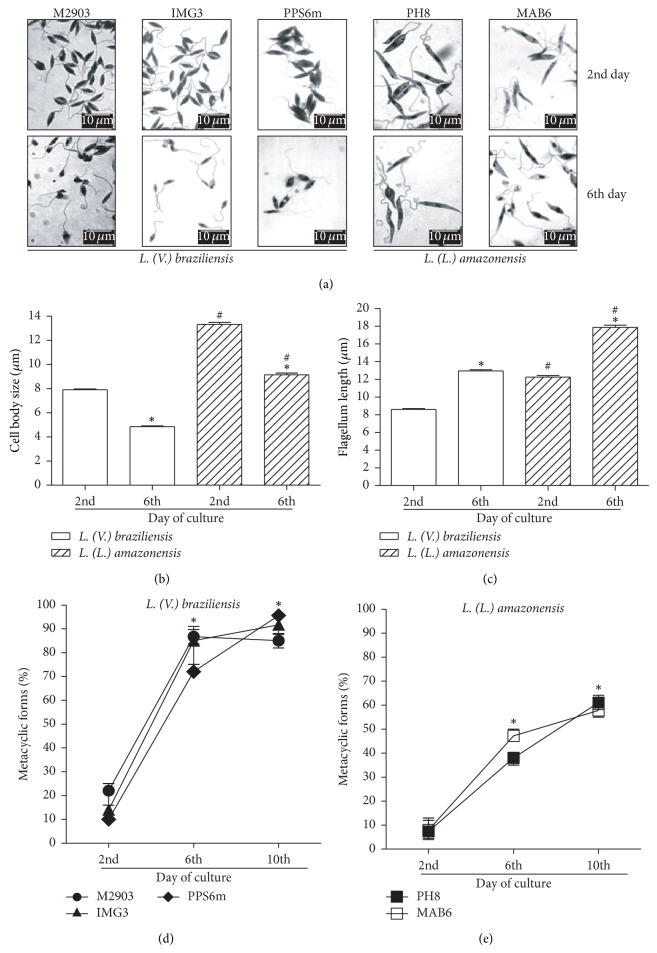
Metacyclogenesis as evaluated by morphometric analysis. Parasites in growth logarithmic phase were distributed into 24-well plates (5 × 10^5^ parasites/mL) in Grace's medium, at 26°C, in triplicates. (a) After 2 or 6 days, parasites (M2903, IMG3 and PPS6m,* L. braziliensis*; PH8 and MAB6,* L. amazonensis*) were fixed on slides, stained, and analyzed under a light microscope (1000x). Bar represents 10 *μ*m. (b) Data show mean ± SEM of cell body size (*μ*m); (c) data show mean ± SEM of flagellum length ((b) and (c)) open bars:* L. braziliensis*; hatched bars:* L. amazonensis*; measurements were obtained with the software image J (*n* = 300 parasites). ^*^ *P* < 0.05 (2nd versus 6th day); ^#^ *P* < 0.05 (*L. braziliensis* versus* L. amazonensis*). (d) and (e) percentages of metacyclic forms (flagellum/body ratio ≥ 2). Data represent median and minimal and maximal values of three independent experiments. ^*^ *P* < 0.05 (2nd versus 6th; 2nd versus 10th day).

We first determined the growth phases of culture by counting parasites each day to establish the growth curves, which were similar among the isolates evaluated (see Figure 1 in Supplementary Material available online at http://dx.doi.org/10.1155/2014/393049). Next, we evaluated the morphological alterations to identify metacyclic forms in cultures.  Figure 1(a) shows different parasite forms at 2nd (logarithmic phase) and 6th days (stationary phase) of culture. From the 2nd to the 6th day of culture, parasites differentiated into forms with smaller cell body (Figures 1(a) and 1(b)) and lengthier flagellum (Figures 1(a) and 1(c)). Morphometric analyses also showed that* L. amazonensis* isolates present larger cell body and lengthier flagellum than* L. braziliensis* isolates (Figures 1(b) and 1(c)). However, no differences in size were detected among isolates from the same species. Morphometric analyses of each isolate at the 2nd, 6th, and 10th days of culture are presented in Supplementary Table 1. At the 6th and 10th days of promastigote culture, ~70 to 90% of promastigotes whose flagellum/body length ratio was ≥2 of* L. braziliensis* (M2903, IMG3 and PPS6m) were detected (Figure 1(d)), whereas only 35 to 50% of* L. amazonensis* (PH8 and MAB6) (Figure 1(e)) were counted.

**Figure 2:**
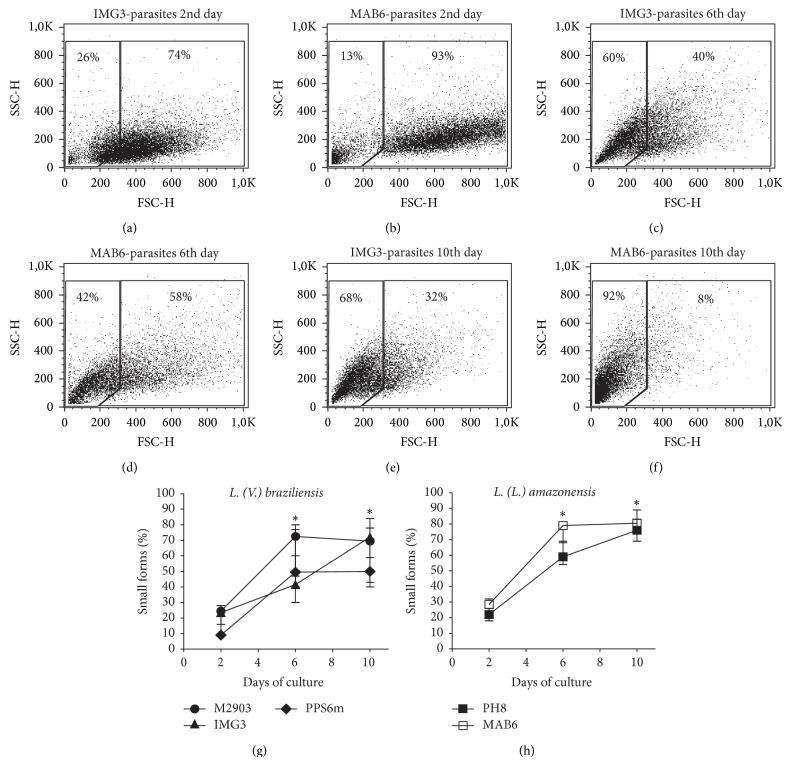
Metacyclogenesis as evaluated by flow cytometry. Parasites in growth logarithmic phase were distributed into 24-well plates (5 × 10^5^ parasites/mL) in Grace's medium, at 26°C, in triplicates. After 2, 6, or 10 day of culture, parasites were analyzed by flow cytometry. Figures show size (FSC) and granulocity (SSC) of parasites from 2nd ((a) IMG3* L. braziliensis*; (b) MAB6* L. amazonensis*), 6th ((c) IMG3; (d) MAB6), and 10th ((e) IMG3; (f) MAB6) days of culture. FSC^low^ are metacyclic forms (left gate) and FSC^high^ are procyclic forms (right gate). (g) and (h) data represent percentage of small forms (median with minimal and maximal values) of four independent experiments. ^*^ *P* < 0.05 (2nd versus 6th; 2nd versus 10th day).

According to Saraiva et al. [11] flow cytometry, based on cell size (FSC), was shown to be a useful tool to identify procyclic (FSC^high^) and metacyclic (FSC^low^) forms.  Figure 2 shows a representative flow cytometry analyses of IMG3 (*L. braziliensis*) and MAB6 (*L. amazonensis*) isolates, at the 2nd (Figures 2(a) and 2(b)), the 6th (Figures 2(c) and 2(d)), and the 10th days (Figures 2(e) and 2(f)) of culture. As observed, the percentage of smaller forms (FSC^low^, left gate) increased along culture time. High frequencies of smaller forms were detected at 6th and 10th days of cultures of both* L. braziliensis* (Figure 2(g)) and* L. amazonensis* (Figure 2(h)) isolates. No differences were found among percentage of smaller forms detected in cultures of different isolates in each evaluated day.

### 3.2. *In Vitro* Metacyclogenesis of Clinical Field Isolates of* L. braziliensis* and* L. amazonensis* as Evaluated by Parasite Surface Modifications (Loss of BPL- and 3A1-La mAb-Binding Ability, Resp., and Acquisition of Complement Resistance)

**Figure 3:**
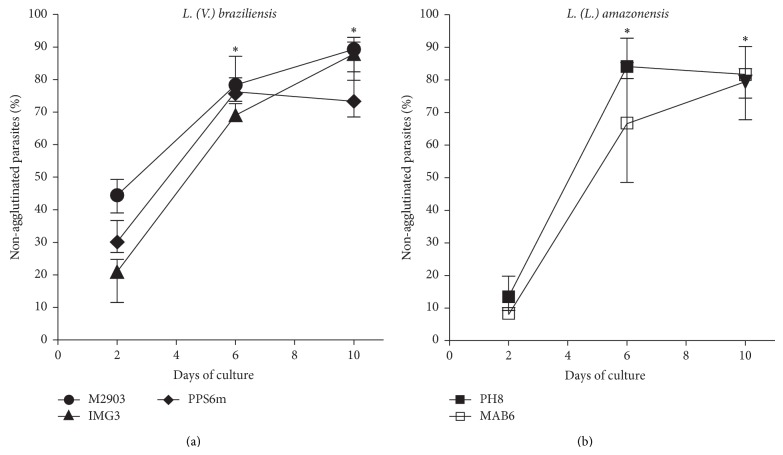
Metacyclogenesis as evaluated by negative selection with lectin or mAb. Parasites in growth logarithmic phase were distributed into 24-well plates (5 × 10^5^ parasites/mL) in Grace's medium, at 26°C, in triplicates. After 2, 6, or 10 days, parasites were incubated with BPL ((a),* L. braziliensis*) or 3A1-La mAb ((b),* L. amazonensis*), and nonagglutinated parasites were considered metacyclic forms. Data represent median and minimal and maximal values of percentages of nonagglutinated forms from 3 independent experiments. ^*^ *P* < 0.05, 2nd versus 6th day; 2nd versus 10th days.

Besides morphological modifications, biochemical alterations also occur in promastigotes during metacyclogenesis. One type of such alterations is the loss of certain carbohydrates in the parasite LPG, which allow the identification/enumeration and selection of metacyclic forms by using lectins or mAbs specific to these carbohydrates present in their LPG [17, 18]. Thus, BPL, a lectin that binds to *β*-gal (1–3)-GalNAc present in LPG of* L. braziliensis* procyclic promastigotes, and the mAb 3A1-La, which binds to* L. amazonensis* procyclic forms, was used here to identify and try to purify the metacyclic promastigotes. Similarly to what we found using the morphology criterion, a significant increase of nonbinding forms from* L. braziliensis* and* L. amazonensis* isolates occurred with the negative selection with BPL (Figure 3(a)) or mAb 3A1-La (Figure 3(b)), respectively, at 6th and 10th days of culture. The percentages of nonagglutinated forms were similar among all isolates assessed.

**Figure 4:**
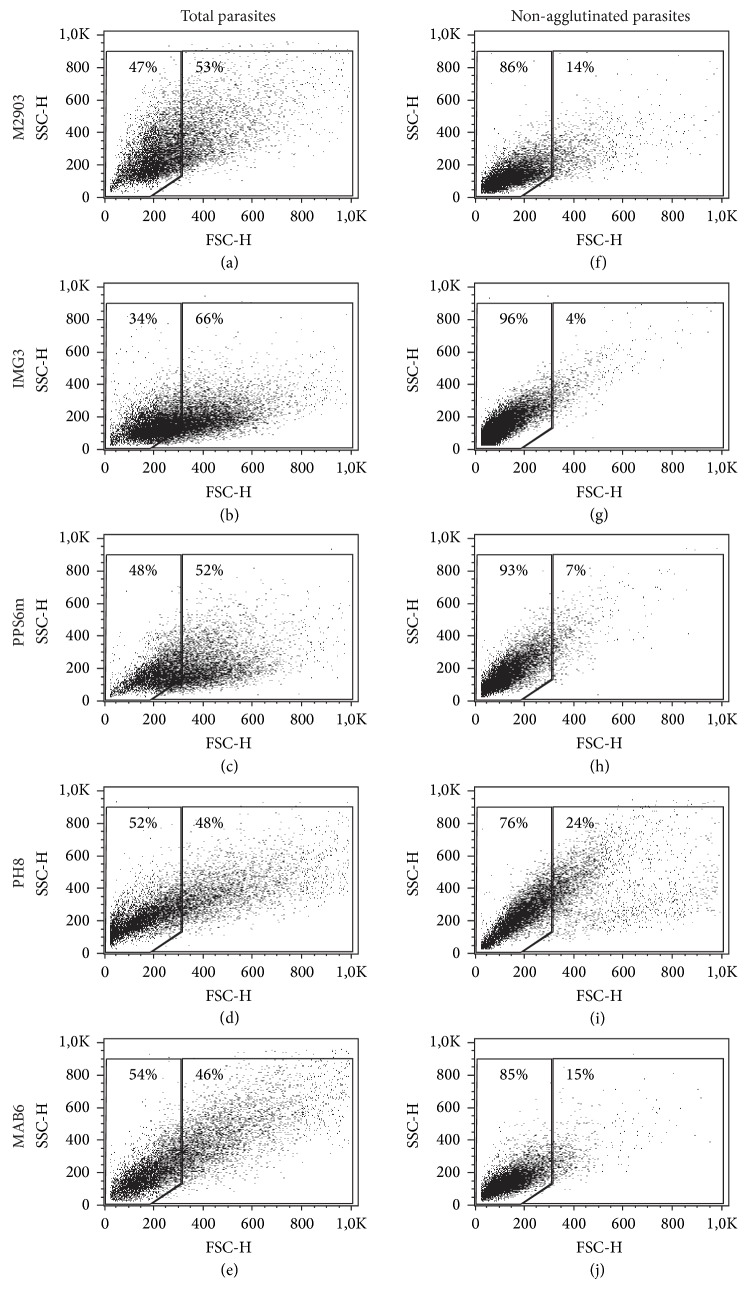
Flow cytometry analyses of BPL- or 3A1-La mAb-negative selected parasites. Nonfractioned parasites (total parasites: (a)–(e)) or negative-selected parasites (nonagglutinated parasites: (f)–(j)) were evaluated by flow cytometry according to FSC × SSC, at 6th day of culture. (a) and (f) M2903; (b) and (g) IMG3; (c) and (h) PPS6m; (d) and (i) PH8; (e) and (j) MAB6. FSC^low^ are small forms (left gate; enriched in metacyclic forms); FSC^high^ are large forms (right gate; enriched in procyclic forms).

As expected, the nonagglutinated promastigotes isolated by negative selection were mostly the small forms as seen by the enrichment in FSC^low^ forms in flow cytometry.  Figure 4 shows a representative experiment with each isolate on the 6th day of culture. In all suspensions of negative selected parasites (independent on the day of culture), flow cytometry showed 75–97% of FSC^low^ forms (data not shown).

**Figure 5:**
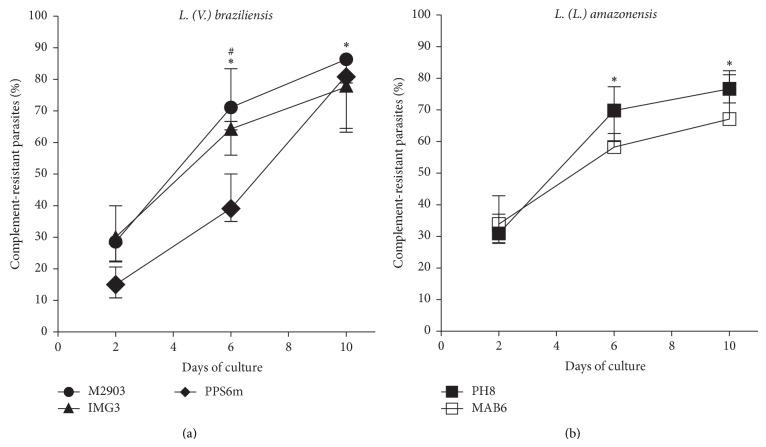
Metacyclogenesis as evaluated by complement resistance. Parasites in growth logarithmic phase were distributed into 24-well plates (5 × 10^5^ parasites/mL) in Grace's medium, at 26°C, in triplicates. After 2, 6 or 10 days of culture parasites were incubated with 10% of rabbit serum and percentage of viable parasites (complement-resistant forms) was determined after 1 h of incubation, at 37°C. Data represent median and minimal and maximal values from 3 independent experiments. ^*^ *P* < 0.05 (2nd versus 6th; 2nd versus 10th day). ^#^ *P* < 0.05 (PPS6m versus M2903/IMG3, 2nd and 6th days).

It has been shown that* L. major* and* L. amazonensis* [30, 31] metacyclic forms present higher resistance to complement lysis due to LPG and gp63 alterations during metacyclogenesis. Here, we show that* L. braziliensis* and* L. amazonensis* change in LPG composition during* in vitro* metacyclogenesis. Thus, parasite isolates were also assayed for complement resistance to compare the percentage of resistant forms in the cultures (2, 6, and 10 days of culture). Likewise, we observed that the resistant forms were much more frequent at the stationary phase of parasite growth (6th and 10th days) than at logarithmic phase (2nd day) (Figures 5(a) and 5(b)). PPS6m isolate, at the 2nd and 6th days of culture, presented lower percentage of complement-resistant forms than the other two isolates but achieved similar percentages at the 10th day (Figure 5 and Supplementary Table 2).

### 3.3. Comparison of the Different Criteria to Evaluate* In Vitro* Metacyclogenesis of Clinical Field Isolates of* L. braziliensis* and* L. amazonensis*

**Figure 6:**
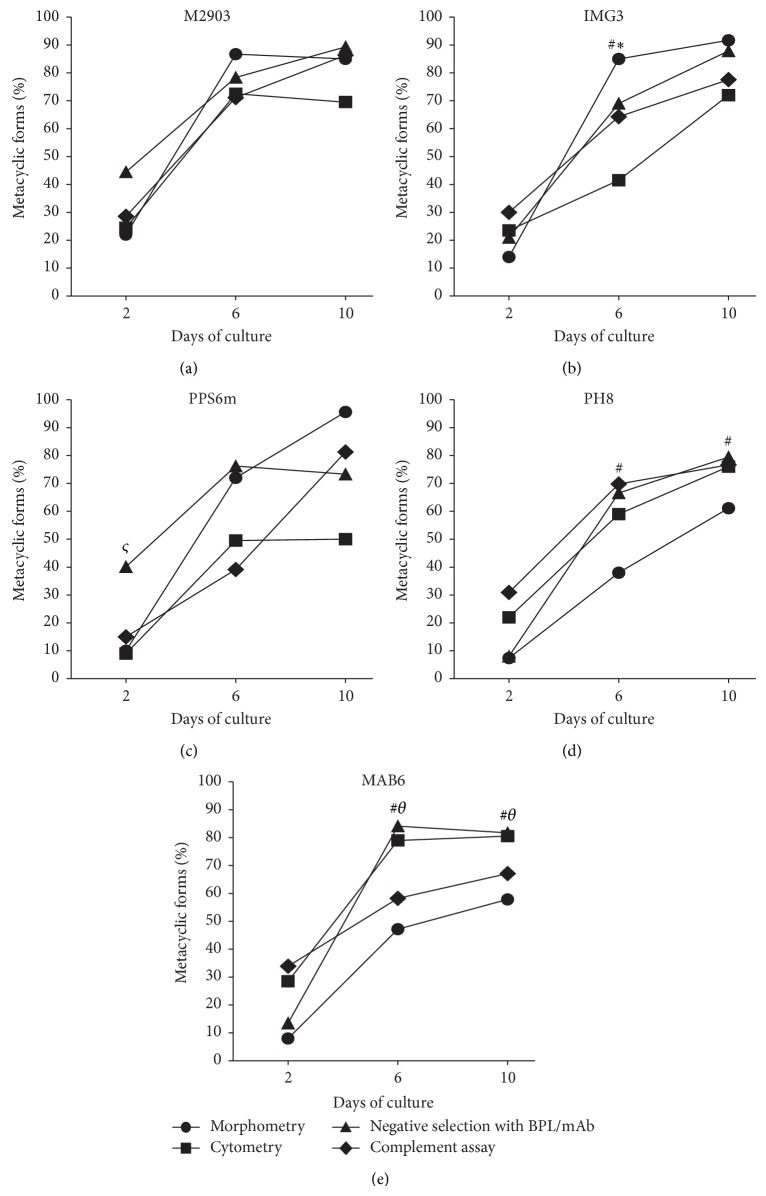
Comparison among the percentages of metacyclic forms detected by morphometry, flow cytometry, negative selection with lectin or mAb, and complement resistance assay. Parasites were cultured and analyzed for metacyclic presence as described in Section 2. Data represent median of percentage of metacyclic forms detected by morphometry (● parasites with flagellum/body size ratio ≥2), flow cytometry (■  FSC^low^ parasites), negative selection with lectin/mAb (▲ negative selected parasites with BPL or 3A1-La mAb), and complement resistance assay (◆ viable parasites after incubation with 10% rabbit serum for 1 h). Parasites were evaluated after 2, 6, or 10 days of culture. (a) M2903; (b) IMG3; (c) PPS6m; (d) PH8; (e) MAB6. Morphometry, negative selection and complement resistance assay were done simultaneously with parasites from the same cultures (*n* = 3 independent experiments in triplicates); flow cytometry was performed separately (*n* = 4 independent experiments in triplicates). ^*^ *P* < 0.05 (Flow cytometry versus other assays); ^#^ *P* < 0.05 (morphometry versus other assays); ^*ς*^ *P* < 0.05 (negative selection versus other assays); ^*θ*^ *P* < 0.05 (complement resistance assay/morphometry versus flow cytometry and negative selection).

Since metacyclogenesis is accompanied by several morphological and biochemical changes, we sought to compare these modifications in the isolates and reference strains studied. It is noticeable that the different features acquired by promastigotes during* in vitro* culture, which are used as criteria to evaluate metacyclogenesis, may develop with different time courses. A comparison among percentages of metacyclic promastigotes detected by the techniques used here is presented in Figure 6. For the reference strain M2903 of* L. braziliensis*, the development of all features has reached its maximum at the 6th day. For the others, however, at least one feature developed slower than others. The morphometric criterion did not seem to go together with the negative selection or cytometry in PH8 and MAB6, whereas in IMG3, size by cytometry, and in PPS6m, size by cytometry and complement resistance were the criteria that differed from size by morphometry and negative selection.

### 3.4. *In Vitro* Metacyclogenesis of Clinical Field Isolates of* L. braziliensis* and* L. amazonensis* as Evaluated by Infectivity to Human Macrophages

**Figure 7:**
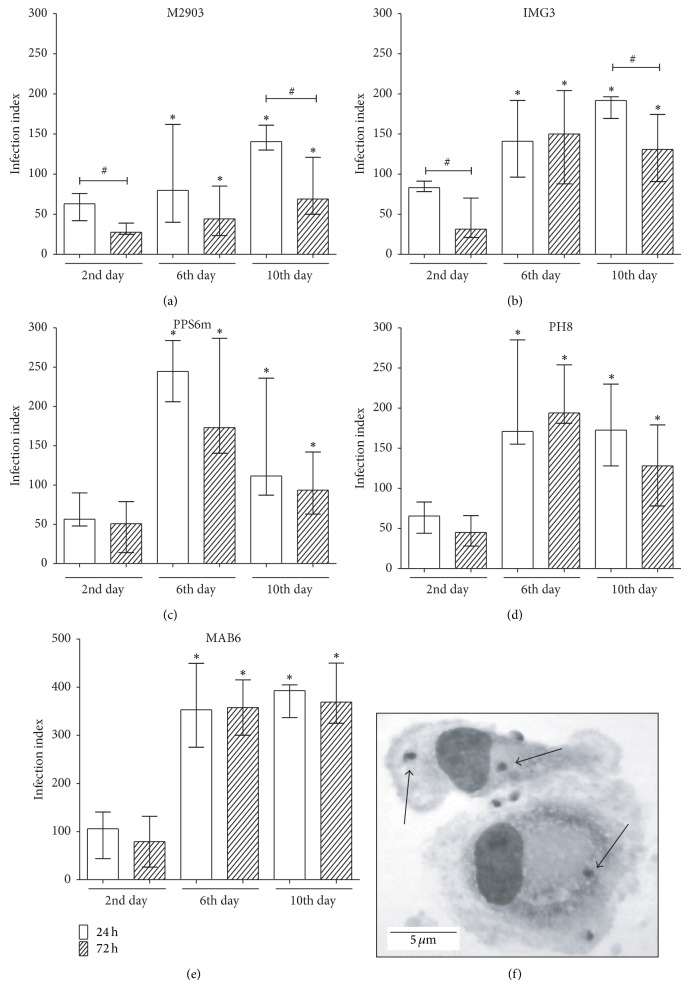
Metacyclogenesis as evaluated by infectivity to human macrophages. Monocyte-derived macrophages were incubated with parasites from different days of culture (2nd, 6th, or 10th). After 24 or 72 h of incubation, cells were stained and evaluated under a light microscope (1000x). (a) M2903; (b) IMG3; (c) PPS6m; (d) PH8; (e) MAB6. Data show median and minimal and maximal values from 6 donors from 2 independent experiments done in triplicates. ^*^ *P* < 0.05 (2nd versus 6th day; 2nd versus 10th day; 24 h; 72 h); ^#^ *P* < 0.05 (24 h versus 72 h). (f) Photomicrography of infected macrophages (IMG3* L. braziliensis*). Arrows indicate amastigotes forms, and bar corresponds to 5 *μ*m.

As showed in Figure 7, promastigotes from stationary phase of growth (6th or 10th day of culture) are more infective than parasites from logarithmic phase (2nd day of culture), as observed by the infection index of macrophages after 24 h or 72 h of infection. The increase of infection index reflects the increase in both the percentage of infected cells and the mean number of parasites per cell (data not shown). These data correlates with both the morphological and biochemical criteria of metacyclogenesis, as described by Pinto-Da-Silva et al. [17], although in the 6th day some of markers of metacyclogenesis for some isolates or reference strains have not reached their top percentage (best seen in Figures 1(e), 2(g), 2(h), 5(a), and 6(b)–6(e)).

It is interesting to note that the infection index decreased from 24 h to 72 h in the cultures with M2903 and IMG3 isolates, especially when parasites were from 2nd or 10th day of culture (Figures 7(a) and 7(b)). The other isolates presented similar levels of infection after 24 h or 72 h regardless of the stage that the parasites were on (Figures 7(c), 7(d), and 7(e)).  Figure 7(f) shows a photomicrography of human macrophages infected with the IMG3 isolate after 24 h of infection.

Parasites submitted to negative selection with BPL or mAb before infecting macrophages showed no differences in macrophage infection indexes compared with total parasites or metacyclic-enriched suspensions (Supplementary Figure 2).

**Figure 8:**
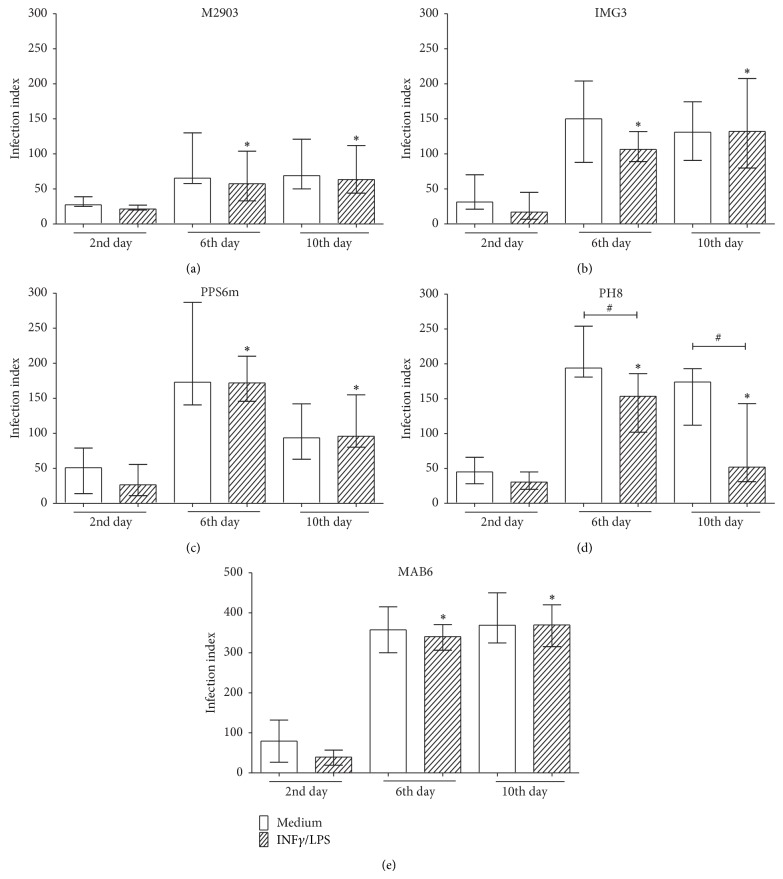
Susceptibility/resistance of parasites to microbicidal activity of classically activated macrophages. Monocyte-derived macrophages were incubated in absence or presence of human rIFN*γ* (10 ng/mL) for 24 h; then parasites from different days of culture (2nd, 6th, or 10th) were added. rIFN*γ*-treated macrophages were also treated with LPS (50 ng/mL). After 72 h of incubation, cells were stained and evaluated under a light microscope (1000x). Data show median and minimal and maximal values from 6 donors in 2 independent experiments. ^*^ *P* < 0.05 (2nd versus 6th day; 2nd versus 10th day - Medium or IFN*γ*/LPS); ^#^ *P* < 0.05 (medium versus IFN*γ*/LPS).

Next, we tested the resistance of parasites to macrophage microbicidal activity induced by a classical activation (priming with human rIFN*γ*, then LPS addition). Similar to that observed with nonactivated macrophages (Figure 7), infection index was higher when parasites from 6th or 10th day were used than parasites from 2nd day of culture (Figure 8). After 72 h of incubation and after macrophage activation with rIFN*γ* and LPS, no further increase in microbicidal activity of macrophages was found against M2903 or IMG3 parasites (Figures 8(a) and 8(b)) or PPS6m and MAB6 (Figures 8(c) and 8(e)), but PH8 strain was better controlled by activated macrophages (Figure 8(d)).

## 4. Discussion

Metacyclogenesis is the process whereby* Leishmania* differentiate from poorly infective procyclic promastigotes into highly infective metacyclic promastigotes. In nature, metacyclogenesis not only occurs in the insect vector but also occurs in axenic culture of promastigotes. In both situations, this differentiation is accompanied by changes in the morphology, including size, shape, and flagellum length, as well as qualitative and quantitative modifications in the expression of surface molecules such as LPG and gp63. Some genes, such as gene B and Mat-1 are tightly linked to metacyclogenesis. Other proteins, Meta-1, SHERP and HASP, were upregulated during the metacyclic stage [8]. The features acquired by promastigotes during metacyclogenesis particularly the biochemical changes have been used as markers of metacyclic promastigotes and are useful to isolate metacyclic from the procyclic forms in a mixed population.

In the present study we evaluated the* in vitro* metacyclogenesis of three clinical field isolates (IMG3 and PPS6m,* L. braziliensis*; and MAB6,* L. amazonensis*). In parallel, known strains of* L. braziliensis* (M2903) and* L. amazonensis* (PH8) were also studied. Our results showed a similar growth profile among all isolates (Supplementary Figure 1), which was also similar to the one previously described for M2903 strain, even when different culture media were used [23, 32]. Since parasite growth rate can vary according to the adaptation of parasites to culture conditions [33], the parasites used here were freshly isolated from mice and cultured for no more than six* in vitro* passages. It has been shown that* L. braziliensis* and* L. amazonensis* in cultures can present similar growth depending on the medium components [32]. Yet, similar growth patterns between* L. braziliensis* and* L. amazonensis* have been described [34]. In the current study, it is possible that the culture conditions (Grace's medium supplemented with 20% FCS) favored the growth and differentiation of both species, allowing all isolate cultures to multiply at similar rates. The similar growth, in fact, facilitated the comparison of metacyclogenesis among all isolates.

The morphological alterations of parasites observed during isolate cultures (Figure 1) were in agreement with those already described for* L. braziliensis* [16, 23] and* L. amazonensis* [18, 29, 35]. Using morphometric analysis we observed that* L. amazonensis *metacyclic forms are larger than* L. braziliensis* (6th and 10th days of culture) (Figures 1(b)–1(e)). Considering the promastigotes whose flagellum/body length ratio was ≥2, the profile of size alteration detected by morphometric analysis was similar among isolates, showing an increase of the percentage of small parasite forms (metacyclic forms) at 6th or 10th day of culture. However, the percentage of* L. amazonensis* promastigotes that fulfilled this criterion was lower than that of* L. braziliensis*, indicating that* in vitro* the relationship body/flagellum size must differ between* L. braziliensis* metacyclic forms and* L. amazonensis* metacyclic forms, which is clearly seen in the images of promastigotes from the 6th day (Figure 1(a)). This suggests that this relationship is not suitable for all species of* Leishmania*.

Also based in morphological alterations, flow cytometry has been used to identify and quantify metacyclic forms. Here, again, using this technique, the profile of metacyclogenesis was similar for all isolates evaluated in this study (Figure 2). The percentage of small parasite forms (metacyclic forms) identified at 2nd and 6th days, as compared to those obtained by Saraiva et al. [11] were similar for* L. braziliensis* (H3456 strain) but different for* L. amazonensis* (Josefa strain), for which we found higher percentages of metacyclic forms. In that case, since the authors evaluated the parasites at 5th day of culture it is possible that a difference of 24 h could be enough to increase the percentage of metacyclic forms detected in the present study (6th day). Although morphometric and cytometric analysis are based on morphological alterations, these techniques detected different percentages of small parasites forms (metacyclic forms) in* L. amazonensis* and also in* L. braziliensis* cultures. It is clear from our experiments that cultures of* L. braziliensis* present more clusters of parasites than* L. amazonensis* which can be detected as large forms in flow cytometry. In addition,* L. amazonensis* presents less alteration of body size than* L. braziliensis* (Figure 1). Thus, our results suggest that depending on the* Leishmania* species, differences in morphology and culture characteristics can influence the ability of different techniques to detect metacyclic forms.

During metacyclogenesis parasites present modifications in LPG carbohydrates allowing them to be isolated by lectin or specific mAb procyclic agglutination assays [9, 17, 18, 36]. Here, the percentage of nonagglutinated parasites increased in parallel with morphological alterations, showing similar profiles of metacyclogenesis for all isolates (Figure 3). Cytometric assessment showed percentages of nonagglutinated parasites similar to those detected by Saraiva et al. [11] (Figure 4). The cytometric analyses of isolated parasites can be useful to check the percentage of metacyclic forms after selection using lectin or mAb. Nevertheless, in the present study suspensions of selected nonagglutinated forms presented 75%–97% of FSC^low^ forms as detected by flow cytometry, suggesting that LPG and morphological alterations are not in complete consonance.

Another characteristic of metacyclic forms, also related to LPG, gp63 and probably other protein modifications, is the increase of parasite resistance to the complement system [7, 13, 14]. In the present study, parasites from both species showed greater resistance to complement in stationary (6th and 10th days of culture) than those from logarithmic phase of growth (2nd day of culture) (Figure 5). These data corroborate those of Almeida et al. [16] and Pinto-Da-Silva et al. [18]. For PPS6m (*L. braziliensis*) and MAB6 (*L. amazonensis*) isolates the percentages of complement-resistant and BPL- or mAb-nonagglutinated forms did not match at 6th-day cultures (Figure 6). These results suggest that even using two techniques based in detection of LPG alterations it is possible that the complement resistance and loss of carbohydrates are not simultaneous events during* in vitro* metacyclogenesis. Based on the results of these two clinical isolates (PPS6m and MAB6) it can be suggested that complement resistance can be acquired hours/days after carbohydrate alterations, suggesting that other factors/molecules not analyzed here are involved.

One important reason to study* in vitro* metacyclogenesis of different clinical field isolates of* Leishmania*, besides understanding the parasite biology, is to establish optimal conditions to investigate the infection by* Leishmania*, particularly the human macrophage infection, since most studies are carried out using the murine model. Our results showed that parasites from 6th or 10th day of culture were more efficient to infect macrophages than promastigotes forms from 2nd day (Figure 7). These results indicate that, indeed, metacyclic forms are present in high frequency in the stationary phase of parasite growth and in low frequency in the logarithmic phase of growth, which correlates with the development of the other features studied here. Despite the similar profile of metacyclogenesis among the isolates, it was observed that in macrophages infected with M2903 or IMG3 parasites (*L. braziliensis*) there was a significant decrease in infection index from 24 h to 72 h of incubation when macrophages were infected with parasites from the 2nd or 10th day of culture. These results suggested that 2nd-day promastigotes could not be well adapted to survive in macrophages and those from 10th day are weakened because of nutrient starvation. In both cases parasites were more susceptible to microbicidal activity of macrophages. One possibility is that parasites from the 2nd day activate macrophages during the first hours of infection inducing microbicidal mechanisms whereas those from 6th day silently infect macrophages. Ueno et al. [37] have showed that* Leishmania* parasites from logarithmic phase of growth, instead of metacyclic forms, use mannose receptor during macrophage phagocytosis activating parasite killing mechanisms. Macrophages infected with 6th-day parasites did not present significant increase of the infection index until 72 h, suggesting that human macrophages allow the infection but do not favor parasite growth. Likewise, Hsiao et al. [38] have demonstrated that primary human macrophage inhibits the growth of* L. infantum chagasi* until 72 h after* in vitro* infection with metacyclic forms. Thus, our results suggest that the infection with* L. braziliensis* or* L. amazonensis* can be controlled in human macrophages during 72 h. Although the mechanisms of parasite control need to be investigated, it has been shown that reactive oxygen species (ROS) are responsible for controlling New World* Leishmania* infection in human macrophages [25, 39].

In the present study, negative selection by agglutination assay was used to obtain selected metacyclic forms and to compare infection of human macrophages with parasites in stationary phase of growth (total parasites, 6th day of culture) or metacyclic forms (Supplementary Figure 2). No significant differences were found in macrophage infection index with total parasites or selected metacyclic forms, either after 24 h or 72 h. This result confirms that the majority of promastigotes on the 6th day of culture is metacyclic, which is also strengthened by the other features analyzed here. Also, after 6 days of culture low amounts of parasites can be procyclic forms and parasites that are differentiating into metacyclic forms could be able to infect macrophages as well as metacyclic forms. Moreover, the isolation of metacyclic forms by negative selection does not ensure 100% efficiency and it is possible that the process only enriches the population in metacyclic forms, so that a low percentage of procyclic forms may be present in the lectin or mAb nonagglutinated population. Thus, due to a continuous* in vitro* differentiation process it can be not possible to detect differences in the outcome of human macrophage infection with stationary phase parasites or selected metacyclic forms of New World species of* Leishmania*. Pinto-Da-Silva et al. [17] showed that BPL-non-agglutinated* L. braziliensis* (cultures with 50% of metacyclic forms detect by this technique) were more infective to murine macrophages than parasites from stationary phase of growth, but without statistical significance. Together, these results suggest that depending on the number of metacyclic forms present in the parasite preparations and the degree of parasite virulence, to enrich metacyclic forms could be need to obtain high levels of macrophage infection. Still, infection with metacyclic forms enriched by negative selection can induce more homogenous results when evaluating the quality of immune responses during* L. braziliensis* or* L. amazonensis* human macrophage infection.

We also evaluated the ability of parasite isolates to establish an infection in classically activated macrophages (IFN*γ* plus LPS; [40, 41]). Classical activation of macrophages induces ROS/reactive nitrogen intermediates that control infection with different* Leishmania* species [25, 39, 42–44]. In our study, parasites from 6th or 10th day of culture lead to higher infection indexes than parasites from the 2nd day independent on macrophages were treated or not with IFN*γ*/LPS. This treatment reduced the infection index caused by PH8, but not for other isolates (Figure 8), suggesting intrinsic resistance mechanisms of parasite isolates to human macrophage microbicidal activity.

This is the first study on* in vitro* metacyclogenesis of* L. braziliensis* and* L. amazonensis* clinical field isolates using, simultaneously, four well established techniques. Although the metacyclogenesis profile of all isolates was similar, depending on the technique used different density of parasites with features of metacyclic forms were detected, indicating that the different features evaluated do not develop simultaneously. Whereas for both species negative selection assay (with BPL or 3A1-La mAb) was the technique that identified higher percentages of parasites with biochemical characteristics of metacyclic promastigotes, morphometry could identify high amounts of* L. braziliensis* small promastigotes, but not of* L. amazonensis*. On the other hand, flow cytometry detected lower percentage of* L. braziliensis* small forms, but high amounts of* L. amazonensis*. Morphometric and cytometric analysis although based on morphological aspects did not detect the same amounts of metacyclic forms (IMG3, PPS6m, PH8, MAB6), as well as complement resistance and negative selection assays, both detecting LPG alterations, were not in accordance for PPS6m and MAB6 isolates. Our results showed that by using different techniques to evaluate different aspects of metacyclogenesis (morphological and biochemical modifications) different percentages of metacyclic promastigotes can be detected in each isolate culture. These results suggest that a continuous differentiation process from procyclic into metacyclic forms is working* in vitro* providing different stages of parasite differentiation.

Thus, according to our results, for each new clinical field isolate to be studied is important to determine the metacyclogenesis profile by different techniques, and to identify the better day of parasite culture to select metacyclic forms to infect macrophages, especially in the case of low rates of metacyclogenesis. To use the same amounts of metacyclic forms to compare the ability of different parasites to infect macrophages, especially when parasite cultures present huge differences in percentage of metacyclic forms, can be determinant in virulence research.

## 5. Conclusion

In our study, clinical field isolates of* L. braziliensis* and* L. amazonensis* although showing similar time course of metacyclogenesis (peak of metacyclic forms at 6th day of culture) can display different abilities in establishing a successful infection in nonactivated or activated human macrophages. The results suggest that* in vitro* metacyclogenesis is a continuous differentiation process from procyclic into metacyclic forms. Thus, the different features acquired by promastigotes during* in vitro* culture (morphological alterations, loss of lectin binding, and complement resistance), which are used as criteria to evaluate metacyclogenesis, may develop with different time courses.

## Supplementary Material

To stablish the time course profile of all isolates evaluated in the present study, clinical field isolates IMG3, PPS6m (*L. braziliensis*) and MAB6 (*L. amazonensis*) as well as M2903 (*L. braziliensis*) and PH8 (*L. amazonensis*) strains were incubated into Grace's insect medium, at 26°C. Parasites were quantified in hemocytometer every day for 12 days. Figure 1 shows that all parasites presented a similar time course profile of in vitro growth.
The parasite morphometric analysis was performed in the 2nd, 6th or 10th day after starting *L. braziliensis* (IMG3, PPS6m, M2903) and *L. amazonensis* (MAB6 and PH8) in vitro cultures. The morphometry of fixed and stained parasites was done by using the software Image J. As showed in Table 1, length of the parasite cell body decreased whereas length of flagellum increased along the culture time (6th and 10h days of cultures) for all isolates, indicating the differentiation of procyclic (more frequent at the 2nd day of cultures) into metacycic forms.
To compare the methods used to evaluate metacyclogenesis using different criteria, all results were showed in Table 2: morphometry, cytometry, negative selection of metacyclic forms with lectin/mAb and complement resistance assay. Results showed that different features to evaluate metacyclogenesis may develop with different time courses. In this study, this was especially observed with PPS6m isolate.
It is known that metacyclic forms are more infective than procyclic forms, therefore human monocyte-derived macrophages were incubated with non-selected (Total) or selected (non-agglutinated) parasites (M2903, IMG3, PPS6m, PH8, MAB6) from 6th day of culture. The infection index was evaluated after 24 h or 72 h of incubation. Results showed that for those isolates/strains it is not necessary to enrich the parasite suspension in metacyclic forms as the data showed a similarity between infection index using total or selected parasites.
