# Supplementary material for: In Vitro Metacyclogenesis of Leishmania (Viannia) braziliensis and Leishmania (Leishmania) amazonensis Clinical Field Isolates, as Evaluated by Morphology, Complement Resistance, and Infectivity to Human Macrophages
Source: Biomed Res Int. 2015 Jan 28;2015:393049. doi: 10.1155/2015/393049 (PMC4324811; doi:10.1155/2015/393049)
Supplement: Supplementary file 1 — To stablish the time course profile of all isolates evaluated in the present study, clinical field isolates IMG3, PPS6m (L. braziliensis) and MAB6 (L. amazonensis) as well as M2903 (L. braziliensis) and PH8 (L. amazonensis) strains were incubated into Grace's insect medium, at 26°C. Parasites were quantified in hemocytometer every day for 12 days. Figure 1 shows that all parasites presented a similar time course profile of in vitro growth. The parasite morphometric analysis was performed in the 2nd, 6th or 10th day after starting L. braziliensis (IMG3, PPS6m, M2903) and L. amazonensis (MAB6 and PH8) in vitro cultures. The morphometry of fixed and stained parasites was done by using the software Image J. As showed in Table 1, length of the parasite cell body decreased whereas length of flagellum increased along the culture time (6th and 10h days of cultures) for all isolates, indicating the differentiation of procyclic (more frequent at the 2nd day of cultures) into metacycic forms. To compare the methods used to evaluate metacyclogenesis using different criteria, all results were showed in Table 2: morphometry, cytometry, negative selection of metacyclic forms with lectin/mAb and complement resistance assay. Results showed that different features to evaluate metacyclogenesis may develop with different time courses. In this study, this was especially observed with PPS6m isolate. It is known that metacyclic forms are more infective than procyclic forms, therefore human monocyte-derived macrophages were incubated with non-selected (Total) or selected (non-agglutinated) parasites (M2903, IMG3, PPS6m, PH8, MAB6) from 6th day of culture. The infection index was evaluated after 24 h or 72 h of incubation. Results showed that for those isolates/strains it is not necessary to enrich the parasite suspension in metacyclic forms as the data showed a similarity between infection index using total or selected parasites. [file 393049.f1.pdf]

Supplementary Figure 1

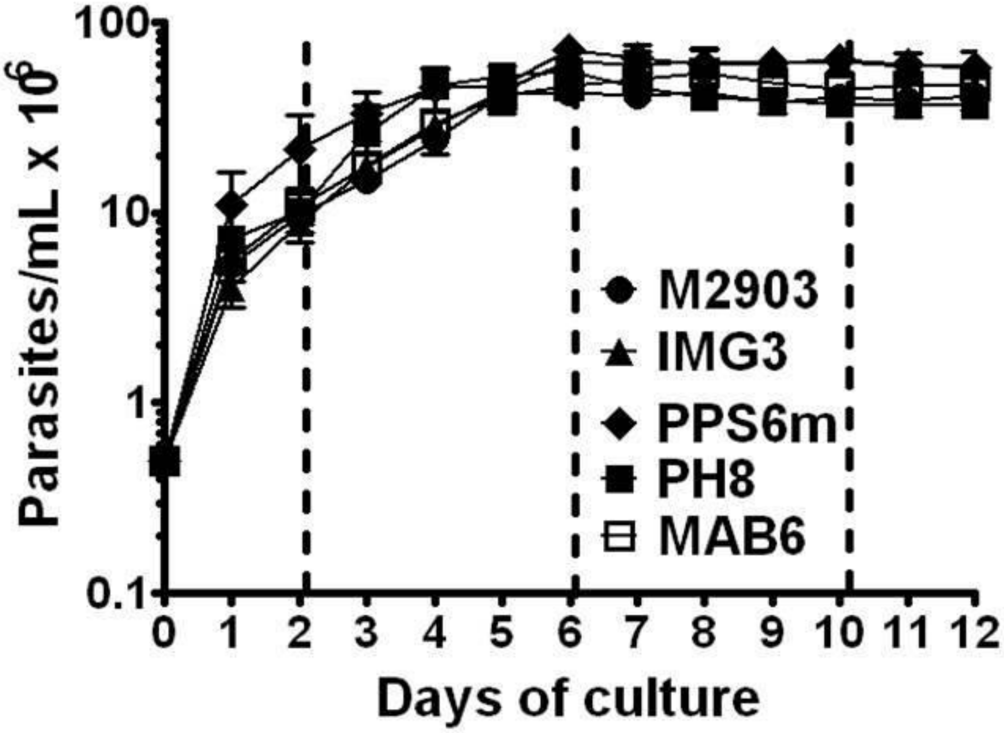

Supplementary Figure 2

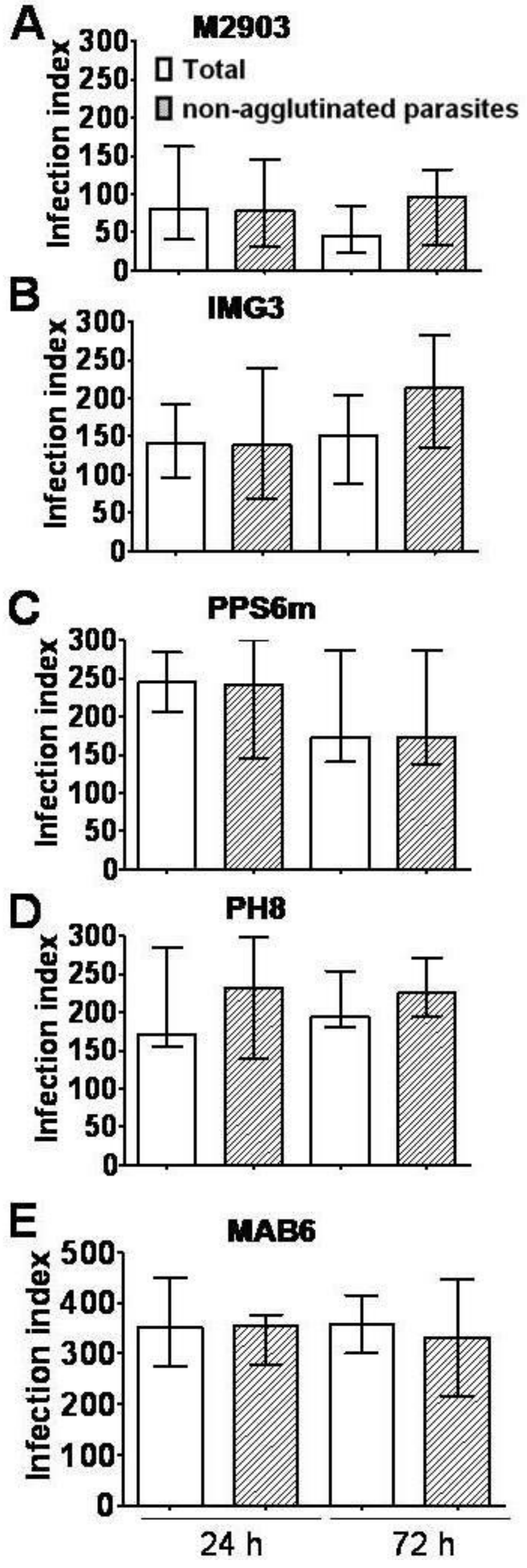

Supplementary Table 1.Morphometry of *L. braziliensis* and *L. amazonensis*promastigotes isolates during *in vitro*growth[a].

*Leishmania*Isolate	Day ofculture	Length of the cell body (µm)	Width of the cell body (µm)	Length of the flagellum (µm)
M2903	2	7.10 ± 0.15	2.23 ± 0.06	8.47 ± 0.23
	6	3.89 ± 0.14*	1.98 ± 0.07	11.03 ± 0.30*
	10	4.53 ± 0.17*	1.84 ± 0.05	12.08 ± 0.37*
IMG3	2	7.06 ± 0.15	1.94 ± 0.05	7.24 ± 0.23
	6	3.71 ± 0.61*	1.97 ± 0.06	12.20 ± 0.34*
	10	3.62 ±0.13*	1.54 ± 0.06	12.38 ± 0.36*
PPS6m	2	7.57 ± 0.09	2.48 ± 0.04	8.94 ± 0.13
	6	3.97 t± 0.06*	2.34 ± 0.04	12.60 ± 0.16*
	10	4.30 ± 0.08*	2.44 ± 0.04	13.74 ± 0.17*
PH8	2	14.74 ± 0.22	2.46 ± 0.07	13.44 ± 0.27
	6	10.62 ± 0.25*	2.66 ± 0.06	20.41 ± 0.40*
	10	8.71 ± 0.16*	2.56 ± 0.06	19.33 ± 0.36*
MAB6	2	11.90 ± 0.20	2.68 ± 0.05	11.06 ± 0.21
	6	7.64 ± 0.16*	2.55 ± 0.07	15.34 ± 0.27*
	10	7.22 ± 0.13*	2.41 ± 0.05	15.43 ± 0.23*

[a]Parasites of *L.* (*Viannia*) *braziliensis*: (IMG3, PPS6m, M2903) and *L. amazonensis* (MAB6 and PH8) were cultured in Grace´s insect medium and after 2, 6 or 10 days parasites were fixed, stained and examined by light microscopy) (1000x) and further analyzed using the software Image J. The values represent mean±EPM of measurements.* P<0.05(2nd*vs* 6th day; 2nd*vs* 10th day); n = 300 parasites/day.

Supplementary Table 2. Percentages of metacyclic forms detected by morphometry, flow cytometry, lectin/antibody selection and complement resistance.

Isolate Day of culture	% of metacyclic forms[a] Morphometry[b]	Citometry[c]	Lectin/Antibody[d]	Complement resistance[e]
**M2903[f] – 2nd Day**	22 [16-25]	24 [16-26]	44 [39-49]	28 [22-40]
**IMG3[g] – 2nd Day**	14 [13-25]	23 [21-28]	20 [11-24]	30 [22-31]
**PPS6m[h] – 2nd Day**	10 [08-12]	09 [07-21]	30 [26-36]	15 [10-20]#
**PH8[i] – 2nd Day**	07 [04-13]	22 [18-26]	08 [07-10]	30 [28-42]
**MAB6[j] – 2nd Day**	08 [03-12]	16 [08-29]	13 [09-19]	33 [27-37]
**M2903 – 6th Day**	86 [83-89]	72 [47-80]	78 [76-87]	71 [64-83]
**IMG3 – 6th Day**	85 [75-91]	41 [30-60]	69 [67-72]	64 [56-66]
**PPS6m – 6th Day**	72 [25-71]	49 [40-77]	76 [75-80]	39 [35-50] #
**PH8 – 6th Day**	38 [35-40]	59 [54-69]	66 [48-85]	69 [60-77]
**MAB6 – 6th Day**	47 [45-50]	79 [68-80]	84 [80-92]	58 [58-62]
**M2903 – 10th Day**	85 [82-88]	69 [59-84]	89 [82-90]	86 [78-86]
**IMG3 – 10th Day**	91 [87-95]	72 [43-78]	87 [79-92]	77 [63-80]
**PPS6m – 10th Day**	95 [93-97]	50 [40-71]	73 [68-91]	81 [64-84]
**PH8 – 10th Day**	61 [58-64]	76 [69-83]	79 [74-83]	76 [72-81]
**MAB3 – 10th Day**	57 [55-60]	80 [78-89]	81 [67-90]	67 [66-82]

[a]Median [range]; [b]parasites that exhibited flagellum/cell body size ratio ≥ 2, [c]parasites FCS^{low}; [d]negatively selected parasites by BPL (*L. (V) braziliensis*) or 3A1-La antibody (*L. amazonensis*); [e]viable parasites after incubation with 10% rabbit serum; [f]reference strain of*L. braziliensis*; [g]Clinical isolate of *L. braziliensis* (LCL); [h]Clinical isolate of *L. braziliensis* (ML); [i]Reference strain of *L. amazonensis*; [j]Clinical isolate of *L. amazonensis* (DCL). #P < 0.05 (PPS6m *vs* all isolates)
